# Genetic Interaction of *Thm2* and *Thm1* Shapes Postnatal Craniofacial Bone

**DOI:** 10.3390/jdb10020017

**Published:** 2022-05-11

**Authors:** Erin E. Bumann, Portia Hahn Leat, Henry H. Wang, Brittany M. Hufft-Martinez, Wei Wang, Pamela V. Tran

**Affiliations:** 1 Department of Dental and Craniofacial Sciences, University of Missouri-Kansas City, Kansas City, MO 64108, USA; plhz72@umkc.edu; 2 Department of Anatomy and Cell Biology, University of Kansas Medical Center, Kansas City, KS 66160, USA; h960w831@kumc.edu (H.H.W.); bjack@kumc.edu (B.M.H.-M.); wwang@stowers.org (W.W.)

**Keywords:** *Ttc21a*, *Ttc21b*, mouse knock-out, jaw, facial bone, viscerocranium, Hh signaling

## Abstract

Ciliopathies are genetic syndromes that link skeletal dysplasias to the dysfunction of primary cilia. Primary cilia are sensory organelles synthesized by intraflagellar transport (IFT)—A and B complexes, which traffic protein cargo along a microtubular core. We have reported that the deletion of the IFT-A gene, *Thm2*, together with a null allele of its paralog, *Thm1,* causes a small skeleton with a small mandible or micrognathia in juvenile mice. Using micro-computed tomography, here we quantify the craniofacial defects of *Thm2^−/−^*; *Thm1^aln/+^* triple allele mutant mice. At postnatal day 14, triple allele mutant mice exhibited micrognathia, midface hypoplasia, and a decreased facial angle due to shortened upper jaw length, premaxilla, and nasal bones, reflecting altered development of facial anterior-posterior elements. Mutant mice also showed increased palatal width, while other aspects of the facial transverse, as well as vertical dimensions, remained intact. As such, other ciliopathy-related craniofacial defects, such as cleft lip and/or palate, hypo-/hypertelorism, broad nasal bridge, craniosynostosis, and facial asymmetry, were not observed. Calvarial-derived osteoblasts of triple allele mutant mice showed reduced bone formation in vitro that was ameliorated by Hedgehog agonist, SAG. Together, these data indicate that *Thm2* and *Thm1* genetically interact to regulate bone formation and sculpting of the postnatal face. The triple allele mutant mice present a novel model to study craniofacial bone development.

## 1. Introduction

Approximately 1/5000 births are affected by a skeletal defect. Most skeletal dysplasias are inherited, and thus, diagnosis, genetic counseling, and therapy are dependent on the underlying genetic basis [1]. For the most part, therapy is limited to surgical procedures that have unpredictable success [2].

Ciliopathies are genetic disorders of primary cilia, which are sensory organelles that are present in most mammalian cells [3,4]. Ciliopathies cause defects in multiple organs, including the skeleton, and a subset of ciliopathies, known as the skeletal ciliopathies, cause osteochondrodysplasias [5,6]. These include Jeune syndrome, oro-facial-digital syndromes, and Sensenbrenner syndrome, which manifest shortened long bones, narrow rib cage, polydactyly, as well as craniofacial defects. Moreover, craniofacial defects also occur in non-skeletal ciliopathies, such as Bardet Biedl Syndrome and Meckel Syndrome [7]. The most common ciliopathic craniofacial defects include micrognathia, cleft lip and/or palate, and variation in midfacial width, including hypertelorism. Other craniofacial defects include midface hypoplasia, flat nasal bridge, low-set ears, prominent forehead, craniosynostosis, macrocephaly, holoprosencephaly, bifid tongue, a thin upper lip, high arched palate, epicanthal folds, and a protruding tongue. These anomalies reflect the importance of primary cilia in forming the face.

Primary cilia mediate multiple signaling pathways and thereby regulate cell behavior, tissue development, and homeostasis. Primary cilia were first demonstrated to regulate the mammalian Hedgehog (Hh) pathway [8,9]. Subsequently, these organelles have been shown to regulate other pathways, including the Wingless Integration Site (Wnt), Transforming Growth Factor Beta (TGF-β), Platelet-Derived Growth Factor Receptor alpha (PDGFRα), Notch, Adenosine Monophosphate-Activated Protein Kinase (AMPK), and multiple cellular processes, including proliferation, differentiation, and autophagy [10,11,12,13,14,15]. The many ciliary-mediated signaling pathways and downstream cellular processes are cell type- and developmental/age-dependent. Many of these same pathways and cellular processes are also required for bone and craniofacial development.

Primary cilia structure and function depend on intraflagellar transport (IFT), which is the bi-directional transport of structural and signaling molecules by protein complexes along a microtubular axoneme [16,17,18]. IFT complex B (IFT-B) proteins, powered by kinesin, transport cargo from the ciliary base to the tip in anterograde IFT, while IFT-A proteins, driven by cytoplasmic dynein, return proteins from the ciliary tip to the base in retrograde IFT. IFT-A proteins also mediate the ciliary entry of membrane and signaling proteins. These functional differences result in contrasting phenotypes in cilia structure and often in signaling pathways in a context-dependent manner [16,19].

Both IFT-B (*Ift80*, *Ift88*, and *Kif3a*) and IFT-A (*Thm1*, *Ift140*, *Ift144*) embryonic or juvenile mouse mutants exhibit polydactyly, shortened long bones, abnormal rib development, and craniofacial defects [20,21,22,23,24]. Specifically, *Ift88*-null, conditional mutant, and hypomorphic mice exhibit midface and mandibular hypoplasia, as well as supernumerary teeth [25,26]. Additionally, the deletion of *Ift88* or *Kif3a* in neural crest cells in mice causes midfacial widening, hypertelorism, micrognathia, and cleft palate [27]. The deletion of *Thm1* in neural crest cells also causes cleft palate. These findings substantiate the importance of functional primary cilia in skeletal and craniofacial development, while underlying mechanisms and functional differences between IFT proteins continue to be challenging to elucidate.

*THM1* (TPR-containing Hh modulator 1; also known as *TTC21B*) encodes an IFT-A protein and Hh modulator [28]. Mutations in *THM1* have been identified in several ciliopathies, including Jeune syndrome, Bardet Biedl Syndrome, and Meckel Syndrome, which manifest with skeletal and/or craniofacial defects [29]. *THM2* (*TTC21A*) is a paralog of *THM1* [28]. In mice, unlike the loss of *Thm1*, the deletion of *Thm2* alone does not cause an overt structural defect. However, the deletion of *Thm2*, together with a null allele of *Thm1*, causes a small skeleton and micrognathia, as well as reduced in vitro bone formation of calvarial-derived osteoblasts [30]. To expand on the role of the *Thm2*; *Thm1* genetic interaction, here we characterize the craniofacial defects in greater detail and investigate the molecular pathways that underlie the osteoblast differentiation defect.

## 2. Materials and Methods

### 2.1. Mouse Generation

*Thm2^−/−^* and *Thm2^+/−^*; *Thm1^aln/+^* mice were generated and maintained on a C57BL6/J background backcrossed 5 generations as described [30]. *Thm2^+/−^*; *Thm1^aln/+^* or *Thm2^−/−^*; *Thm1^+/+^* females were mated with *Thm2^+/−^; Thm1^aln/+^* males to generate *Thm2^−/−^*; *Thm1^aln/+^* triple allele mutant mice. The animal procedures were conducted in accordance with the KUMC-IACUC and AAALAC rules and regulations.

### 2.2. Scanning, Reconstruction, and Landmark Placement

Ethanol-fixed heads from postnatal day (P) 14 control and *Thm2^−/−^*; *Thm1^aln/+^* mice were scanned in a Skyscan 1174 micro-computed tomography (μCT) system (Bruker, Billerica, MA, USA) at 50 kVp, 800 µA, with a resolution of 14 µm^3^. A 0.5 mm aluminum filter was used. The integration time was set to 3000 ms. The scan orbit was 180°/360° with a rotation step of 0.3°.

**Figure 1:**
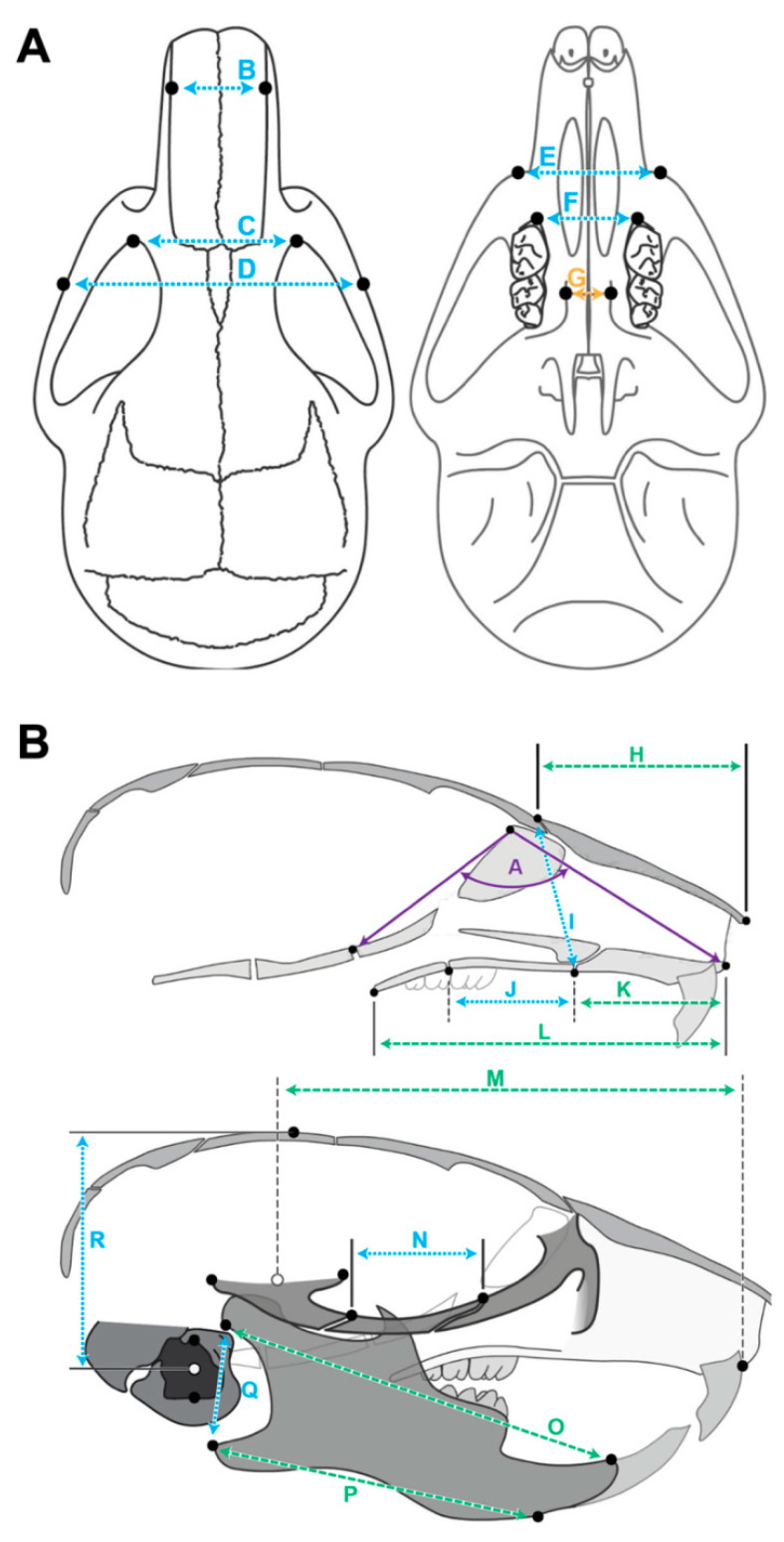
**Schematic of craniofacial measurements and landmarks.** (**A**) Dorsal (left) and ventral (right), as well as (**B**) lateral interior (top) and exterior (bottom) landmarks were placed at the points indicated by black dots. Measurements are represented by double-sided arrows. Measurements that have significant differences between control and *Thm2^−/−^*; *Thm1^aln/+^* mice in true measurements are indicated by dotted blue lines. Measurements that have significant differences between control and *Thm2^−/−^*; *Thm1^aln/+^* mice as both true and cranium centroid measurements are indicated by green dashed lines. The measurement that was significantly wider in *Thm2^−/−^*; *Thm1^aln/+^* mice relative to control mice relative to cranium centroid size is indicated by orange dashed lines. Angular measurement is indicated by solid purple lines. (A) Snout angle, (B) Anterior nasal width, (C) Inter-orbital width, (D) Inter-zygomatic arch width, (E) Inter-maxillary width, (F) Inter-(1st)molar width, (G) Palatal width (H) Nasal length, (I) Facial height, (J) Maxillary length, (K) Premaxillary length, (L) Facial region length, (M) Upper jaw length, (N) Zygomatic length, (O) Mandibular superior and (P) inferior length, (Q) Mandibular posterior height, and (R) Ear height. All analyzed landmarks and measurements, including those not significantly different between control and *Thm2^−/−^*; *Thm1^aln/+^* mice, are provided in Supplemental Table S1.

All data were reconstructed using NRecon software version 1.7.4.2 (Bruker, Billerica, MA, USA) with Gaussian smoothing, ring-artifact reduction, and beam-hardening correction applied. Three-dimensional renderings were created, and landmarks were placed using open-source Drishti software version 2.6.5 (National Computational Infrastructure’s VizLab, Canberra, Australia). Landmarks were placed on the skulls, as shown in Figure 1 and as previously published [31]. The landmarks for ear height were created and placed on the skulls since low-set ears are seen in some patients with primary ciliopathies, and we wanted to determine if low-set ears were present in our *Thm2^−/−^*; *Thm1^aln/+^* mice. The landmarks for ear height were placed at the superior and inferior points on the margin of the external auditory meatus. These landmarks were averaged to determine the center of the ear, and then a measurement was taken from a projected plane intersecting with the top of the skull to a projected plane intersecting with the center of the ear to determine ear height as described [31]. All landmarks were digitized by the same researcher.

### 2.3. Measurements

Euclidean measurements, including the angles and distances between the points were taken for all measurements. Significant measurements are listed in Supplemental Table S1, and significantly altered measurements are depicted in Figure 1. Projected distances between parallel planes intersecting landmarks were determined, and all significant measurements are listed in Supplemental Table S1 and shown in Figure 1. The projected planes were determined as previously described [31]. In short, a transverse projected plane was defined by three points, specifically the anterior distal points of both maxillary first molars and the anterior distal point of one maxillary second molar. A mid-sagittal projected plane was constructed perpendicular to the transverse plane, and an axial projected plane was constructed perpendicular to both the transverse and mid-sagittal planes. For example, in axial-projected landmarks, axial planes parallel to the original axial plane pass through landmarks of interest, and the shortest distance between planes is measured. In transverse-projected landmarks, the same method was used with transverse planes. An average of the right and left measurements was used for all bilaterally paired landmarks.

Since the skulls of the *Thm2^−/−^*; *Thm1^aln/+^* mice are smaller than the skulls of the control mice [30], all measurements were taken and shown as true measurements, as well as adjusted for centroid size. The centroid size of each cranium and mandible was calculated using the root centroid size equation, $RCS=\sqrt{\overset{n}{\underset{i=1}{\sum }}{\left(x_{i}-\bar{x}\right)}^{2}+{\left(y;-\bar{y}\right)}^{2}+{\left(z_{i}-\bar{z}\right)}^{2}}$, where *x*, *y*, and *z* were determined from the coordinates of the 48 Euclidean landmarks of the cranium and 4 Euclidean landmarks of the mandible placed on each microCT 3D reconstruction (Supplemental Table S2). These 52 Euclidean landmarks are composed of single, paired, and the individual of the paired type measurements.

Then each coordinate was divided by cranium centroid size before calculating the distance. This converts the measurements into a ratio of the entire cranium size, which are then compared between groups [32].

### 2.4. Primary Osteoblast Generation and Differentiation Assay

The calvaria of P10 mice were used to generate primary osteoblasts [33]. Briefly, the calvaria were dissected, then digested in αMEM with 2 mM of L-glutamine and 0.2% collagenase, Type I (Sigma-Aldrich, St. Louis, MO, USA T1005)/0.05% trypsin (Sigma-Aldrich, St. Louis, MO, USA C9891) for 20 min at 37 °C in a cell incubator. The media from this first digestion was discarded. An additional 4 rounds of digestion were performed. Following each of these digestions, the media was collected, and fetal bovine serum (FBS) was added to 10% final concentration and kept at 4 °C. Following the 5th digestion, all of the media containing digested calvaria with 10% FBS was filtered through a cell strainer with 70 μm pores. The filtrate was centrifuged, pelleting the cells. Cells were resuspended in αMEM with 10% FBS, 2 mM L-glutamine, and penicillin/streptomycin.

Osteoblasts were plated in 24-well plates in αMEM (Fisher Scientific, Hampton, VA, USA 12-571-063) with 10% FBS, 2 mM L-glutamine, and penicillin/streptomycin. Once the cells reached confluency, differentiation media (αMEM, 10% FBS, 2 mM L-glutamine, penicillin/streptomycin and 50 μg/mL ascorbic acid and 5 mM β-glycerophosphate) was applied to the cells, and refreshed every 3 days over a 21-day period. To examine the effect of SAG, the differentiation media was supplemented with 0.1% DMSO or 500 nM of SAG throughout the 21-day period. Following the differentiation assay, cells were fixed in 10% formalin for 15 min, then immersed in 2% alizarin red solution for 10–15 min in the dark to stain bone nodules. Once the desired color intensity was obtained, cells were washed 4 times with distilled water, immersed in PBS, then fixed in 10% formalin. Wells were imaged using an EVOS^®^ FL Auto system (ThermoFisher Scientific, Waltham, MA, USA) attached to a CMOS color camera.

### 2.5. SAG Treatment and qPCR

Confluent cells (differentiation day 0) or cells differentiated for 7 days were treated with 0.1% DMSO or 500 nM SAG for 48 h or throughout the 7-day differentiation period, respectively. Cells were lysed in Trizol Reagent (Fisher Scientific, Hampton, MA, USA 15-596-026) and stored at −80 °C. Once all of the samples were collected, the RNA was extracted according to the manufacturer’s protocol. cDNA was acquired from the RNA (1 μg) using the Quanta Biosciences qScript cDNA mix (VWR International, Radnor, PA, USA 101414-106). qPCR was performed in duplicate using the Quanta Biosciences Perfecta qPCR Supermix (VWR International, Radnor, PA, USA 101414-120) and a BioRad CFX Connect Real-Time PCR Detection System. Primers used were *Runx2* (Forward: 5′-CCCAGCCACCTTTACCTACA-3′; Reverse: 5′-CAGCGTCAACACCATCATTC-3′) [34]; *Col1α1* (Forward: 5′-GCATGGCCAAGAAGACATCC-3′) [35]; Reverse: 5′-CCTCGGGTTTCCACGTCTC-3′); *Tgf-β3* (Forward: 5′ GGCCAGTTCATTGTGCCCGCC 3′; Reverse: CGGTGATGACCCACGTCCCC 3′) [36]; *Smad3* (Forward: 5′ ACCAAGTGCATTACCATCC 3′; Reverse: 5′ CAGTAGATAACGTGAGGGAGCCC 3′) [35]; *Smad6* (Forward: 5′ ATTCTCGGCTGTCTCCTCCT 3′; Reverse: 5′ CCCTGAGGTAGGTCGTAGAA 3′) [37]; and housekeeping gene *Oaz1* (Forward: 5′-GCC TGA GGG CAG TAA GGA C-3′; Reverse: 5′-GGA GTA GGG CGG CTC TGT-3′) [38].

### 2.6. Statistics

Unpaired, two-tailed *t*-tests for the comparison of two groups and Mann–Whitney tests or one-way ANOVA followed by Tukey’s test for the comparison of more than two groups were used to evaluate statistical significance (*p* < 0.05) using GraphPad Prism, version 9.2.0 (GraphPad, San Diego, CA, USA). The data are presented as scatter plots with mean and standard deviations, with each individual’s values plotted. The effect size was calculated using Excel (Microsoft, Redmond, Washington, DC, USA) using the following equation.

$$Effect\;Size=\frac{\left[Mean\;of\;experimental\;group\right]-\left[Mean\;of\;control\;group\right]}{\left[Standard\;Deviation\;of\;control\;group\right]}$$

## 3. Results

### 3.1. Triple Allele Mutants Have Reduced Skull and Mandibular Measurements

**Figure 2:**
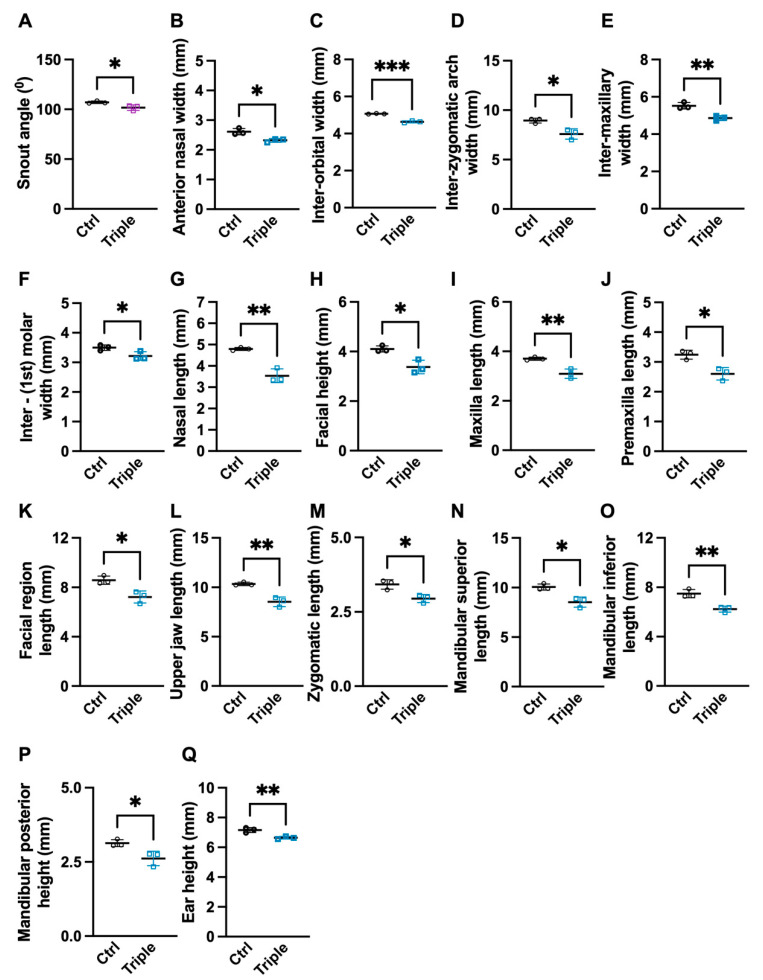
**Significantly smaller true measurement differences in *Thm2^−/−^*; *Thm1^aln/+^* mice.** True measurements of control (Ctrl) and *Thm2^−^^/**−**^*; *Thm1^aln/+^* (Triple) mutant (**A**) snout angle; (**B**) anterior nasal width; (**C**) inter-orbital width; (**D**) inter-zygomatic arch width; (**E**) inter-maxillary width; (**F**) inter-(1st) molar width; (**G**) nasal length; (**H**) facial height; (**I**) maxillary length; (**J**) premaxillary length; (**K**) facial region length; (**L**) upper jaw length; (**M**) zygomatic length; (**N**) mandibular superior length; (**O**) mandibular inferior length; (**P**) mandibular posterior height; (**Q**) ear height. n = 3 control; n = 3 triple allele mutant. Data points represent individual mice. Bars represent mean ± SD. Statistical significance was determined using unpaired *t*-tests. * *p* < 0.05; ** *p* < 0.01; *** *p* < 0.001.

To determine the effect of the loss of *Thm2* together with the *aln* (null) allele of *Thm1* on craniofacial development, we landmarked the µCT skull three-dimensional images of P14 control and *Thm2^−/−^*; *Thm1^aln/+^* littermates. When calculating the Euclidean measurements, including angular and distance measurements, multiple skull measurements were significantly decreased in the *Thm2^−/−^*; *Thm1^aln/+^* mice compared to the control mice (Figure 1 and Figure 2 and Supplementary Table S1). Specifically, *Thm2^−/−^*; *Thm1^aln/+^* mice showed smaller snout angle and multiple smaller distances on the skull, including anterior nasal width, inter-orbital width, inter-zygomatic arch width, intermaxillary width, inter-first molar width, palatal width, facial height, ear height, mandibular posterior height, maxilla length, nasal length, premaxilla length, facial region length, upper jaw length, zygomatic length, mandibular superior length, and mandibular inferior length. Therefore, approximately 60% of the distance measurements analyzed were significantly smaller.

**Figure 3:**
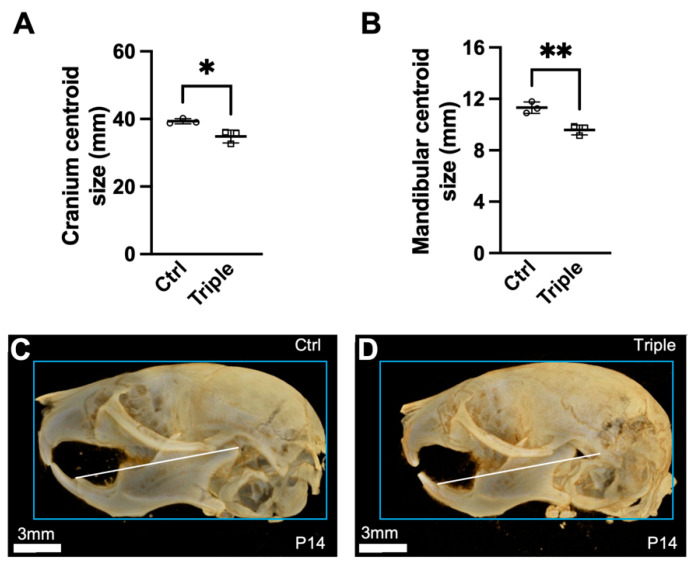
**Centroid size differences in *Thm2^−/−^*; *Thm1^aln/+^* mice.** (**A**) Cranium centroid size and (**B**) Mandibular centroid size. (**C**) MicroCT 3D reconstructions of of control and (**D**) *Thm2^−/−^*; *Thm1^aln/+^* mouse skulls. The blue box (same size) in both images shows the overall cranium size difference and the white line (same length) in both images shows the overall mandibular size difference. n = 3 control; n = 3 triple allele mutant. Data points represent individual mice. Bars represent mean ± SD. Statistical significance was determined using unpaired *t*-tests. * *p* < 0.05; ** *p* < 0.01.

**Figure 4:**
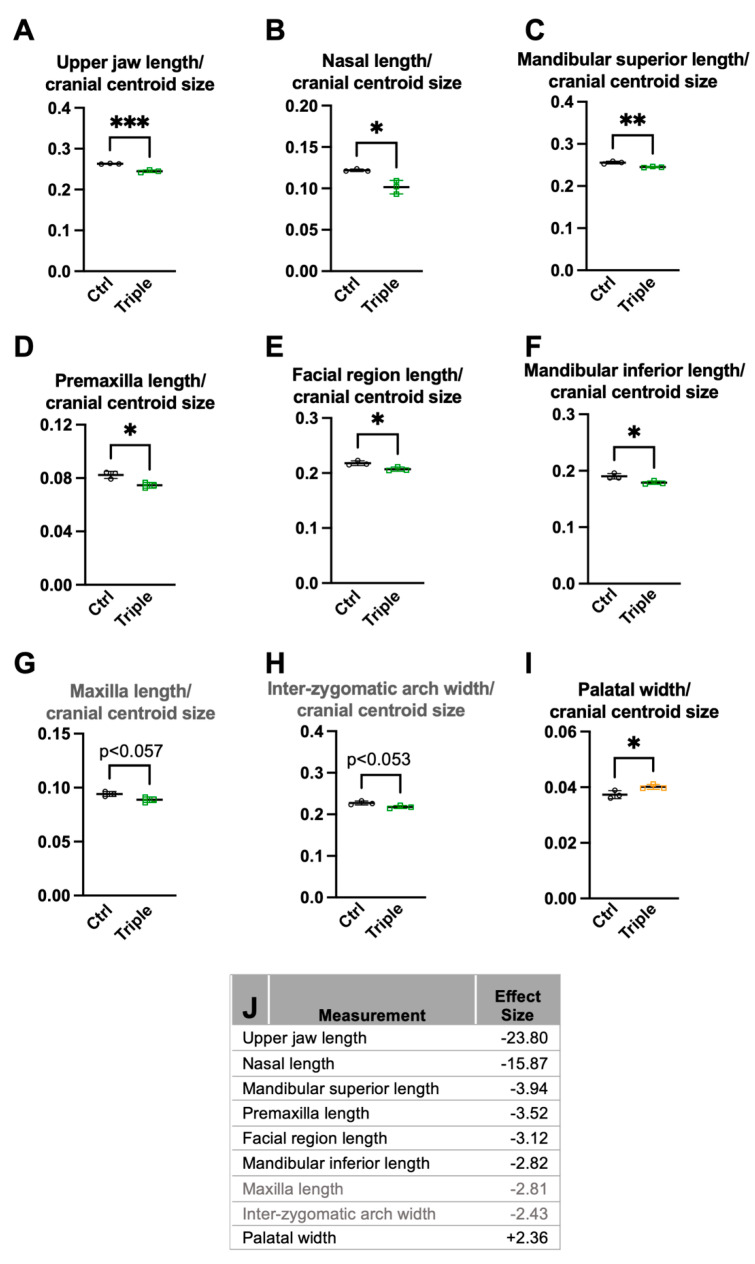
**Significantly smaller measurement differences relative to cranium centroid size in *Thm2^−/−^*; *Thm1^aln/+^* mice.** When correcting for cranium centroid size, triple allele mutant mice have smaller (**A**) upper jaw length; (**B**) nasal length; (**C**) mandibular superior length; (**D**) premaxillary length; (**E**) facial region length; (**F**) mandibular inferior length; (**G**) maxillary length; (**H**) inter-zygomatic arch width; and increased (**I**) palatal width. (**J**) Effect size of measurements in (**A**–**I**). n = 3 control; n = 3 triple allele mutant. Data points represent individual mice. Bars represent mean ± SD. Statistical significance was determined using unpaired *t*-tests. * *p* < 0.05; ** *p* < 0.01; *** *p* < 0.001.

We next analyzed the cranium and mandibular centroid sizes. *Thm2^−^^/**−**^*; *Thm1^aln/+^* mice showed smaller cranium and mandibular centroid sizes than the control mice (Figure 3A,B), consistent with the small mandible phenotype we noted previously [28]. Representative microCT 3D reconstructions illustrate the smaller cranium and mandibular centroids of *Thm2^−^^/**−**^*; *Thm1^aln/+^* mice (Figure 3C,D). Moreover, when correcting for the cranium centroid size, the *Thm2^−^^/**−**^*; *Thm1^aln/+^* mice showed smaller Euclidean distance measurements compared to the control mice, including the facial region length, premaxilla length, nasal length, mandibular superior length, mandibular inferior length, upper jaw length, as well as maxillary length, which approached significance (*p* < 0.057) (Figure 4A–G). Effect sizes for these measurements were negative, indicating the diminution in size (Figure 4J). Reduced distances in these parameters indicate midface hypoplasia and micrognathia in *Thm2^−^^/**−**^*; *Thm1^aln/+^* mice.

Additionally, when correcting for the cranium centroid size, *Thm2^−/−^*; *Thm1^aln/+^* mice showed a significantly wider palatal width compared to the control mice, and the inter-zygomatic arch width approached significant narrowing (*p* < 0.053; Figure 4H,I). The effect sizes for these measurements are noted (Figure 4J). Therefore, approximately 27% of the distance measurements analyzed relative to centroid size were significantly altered.

### 3.2. Hedgehog Agonist Enhances In Vitro Bone Formation of Calvarial-Derived Thm2^−^^/^^−^; Thm1^aln/+^ Osteoblasts

**Figure 5:**
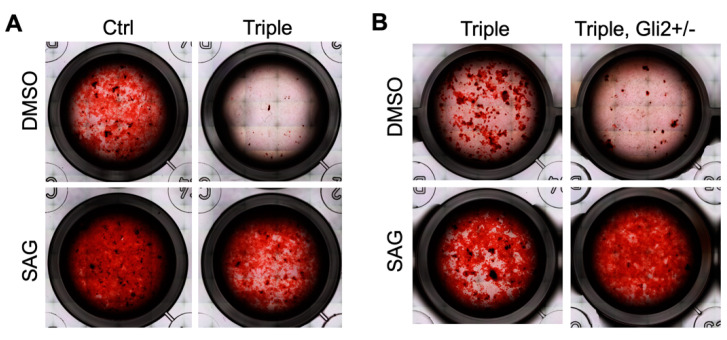
**Amelioration of in vitro bone formation defect in *Thm2^−/−^*; *Thm1^aln/+^* osteoblasts by Hh agonist.** Images of bone nodules on day 21 of an in vitro differentiation assay using calvarial-derived osteoblasts of P10 (**A**) control and *Thm2^−/−^*; *Thm1^aln/+^* littermates and (**B**) *Thm2^−/−^*; *Thm1^aln/+^* and *Thm2^−/−^*; *Thm1^aln/+^*; *Gli2^+/−^* littermates. Media was supplemented with either DMSO (control) or Hh agonist (SAG) throughout the differentiation assay. n = 4 control, n = 4 triple, n = 2 triple; *Gli2^+/−^* from 3 litters.

Using an in vitro osteoblast differentiation assay in which the osteoblasts are differentiated to form bone nodules, we previously observed that triple allele mutant osteoblasts derived from calvaria have reduced bone formation [30]. We have also observed that deletion of one allele of *Gli2* creates and exacerbates the small skeleton phenotype in *Thm2^−/−^* and *Thm2^−/−^*; *Thm1^aln/+^* juvenile mice, respectively, suggesting that the loss of *Thm2* sensitizes bone development to the Hedgehog (Hh) pathway [30]. We, therefore, assessed the roles of *Gli2* and of Hh agonist, SAG, on the in vitro differentiation of *Thm2^−/−^*; *Thm1^aln/+^* calvarial-derived osteoblasts. As expected, the formation of bone nodules by the triple allele mutant cells was reduced at differentiation day 21. However, the treatment with SAG enhanced the bone formation of both the control and triple allele mutant cells (Figure 5A). Additionally, consistent with *Gli2* deficiency exacerbating the triple allele mutant small phenotype in vivo [30], the loss of one allele of *Gli2* in triple allele mutant cells worsened the in vitro bone formation defect, which was ameliorated by SAG (Figure 5B). These data indicate that increased Hh signaling can offset the impaired bone formation of *Thm2^−/−^*; *Thm1^aln/+^* cells.

**Figure 6:**
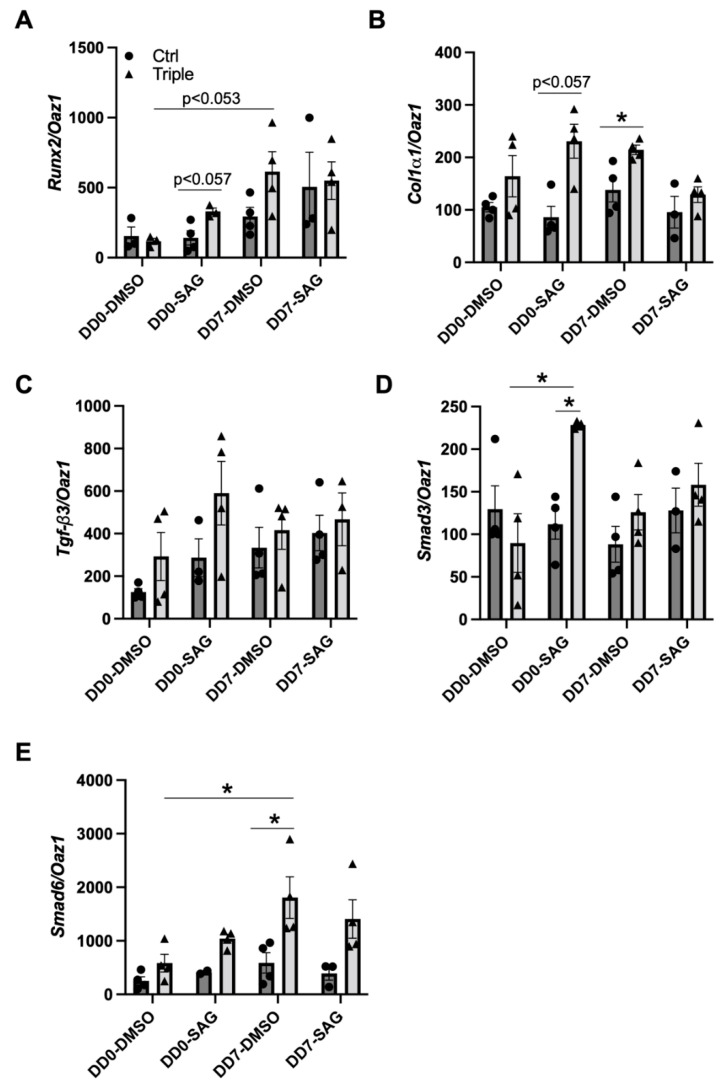
**Transcripts of *Thm2^−/−^*; *Thm1^aln/+^* calvarial-derived osteoblasts.** qPCR for (**A**) *Runx2*; (**B**) *Col1α1*; (**C**) *Tgf-β3*; (**D**) *Smad3*; (**E**) *Smad6*, using RNA extracts of P10 control and *Thm2^−/−^*; *Thm1^aln/+^* calvarial-derived osteoblasts at differentiation day 0 (DD0) or DD7 treated with either DMSO or SAG. n = 4 control, n = 4 triple allele mutant from 3 litters. Data points represent individual mice. Bars represent mean ± SEM. Statistical significance was determined using Mann-Whitney tests for comparison between control and triple allele mutant samples within a treatment group and using one-way ANOVA followed by Tukey’s test for comparison between treatment groups for control or triple allele mutant samples, respectively. * *p* < 0.05.

To determine molecular mechanisms underlying the triple allele mutant in vitro bone formation defect, we analyzed transcript levels of early osteoblast differentiation markers, *Runx2* and *Col1α1*, as well as components of the TGF-β signaling pathway, including *Tgf-β3*, *Smad3*, and *Smad6,* which is an inhibitory molecule of the pathway. Over 7 days of differentiation, triple allele mutant cells showed a trend toward increased *Runx2* (Figure 6A). Additionally, *Col1α1* was increased in the triple allele mutant cells relative to control cells at differentiation day (DD) 7 (Figure 6B). The addition of SAG to the triple allele mutant cells caused an immediate trend toward increased *Runx2* and *Col1α1* and increased *Smad3* relative to control cells (DD0), an effect that did not continue to DD7 (Figure 6A–D). From DD0 to DD7, inhibitory *Smad6* increased and was elevated at DD7 in triple allele mutant cells relative to the control cells (Figure 6E). The addition of SAG from DD0 to DD7 reduced this effect. Taken together, these data suggest that prior to differentiation, triple allele mutant osteoblasts have a heightened response to SAG. Additionally, despite some differences in *Col1α1* and *Smad6* transcript levels, the lack of reduction in any of the transcripts examined suggests that the early stages of osteoblast differentiation in triple allele mutant cells are largely intact.

## 4. Discussion

Osteogenesis is a multi-step process that involves induction, proliferation, condensation, differentiation, matrix (osteoid) deposition, vascularization, mineralization, and remodeling. Two types of ossification—intramembranous and endochondral—develop the skeleton. During intramembranous ossification, mesenchymal progenitor cells differentiate directly into osteoblasts, which then mature into osteocytes. In contrast, during endochondral ossification, mesenchymal progenitor cells differentiate into chondrocytes, which undergo maturation before being replaced by invading osteoblasts. While intramembranous ossification develops most bones of the cranial vault and facial skeleton, including the maxilla and distal mandible, endochondral ossification develops most of the remaining skeleton, including the long bones, the cranial base, and proximal mandible [39]. Micrognathia in triple allele mutant mice may reflect that intramembranous and/or endochondral ossification may be disrupted [40]. In support of an endochondral ossification defect, we have previously found that the *Thm2^−/−^*; *Thm1^aln/+^* mice show a chondrocyte differentiation defect in the tibia [30]. However, since intramembranous and endochondral ossification converge on osteoblasts, it is also possible that the micrognathia in *Thm2^−/−^*; *Thm1^aln/+^* mice may also result from an osteoblast differentiation defect.

Once mesenchymal cells in intramembranous ossification and osteo-chondro progenitor cells in endochondral ossification commit to the osteoblast lineage, osteoblast differentiation can be described in three stages based on the cell cycle stage and gene expression [40]. *Runx2* expression is required for the progenitors to commit to the osteoblast lineage. Subsequently, in the first stage of differentiation, osteoblasts proliferate and express collagen type I, TGF-β receptors, and osteopontin. In the second stage of differentiation, osteoblasts exit the cell cycle and express alkaline phosphatase and collagen type I for the maturation of the extracellular matrix. In the third stage of differentiation, mature osteoblasts secrete osteocalcin and mineralize the extracellular matrix. Although some variations in transcript levels of early osteoblast markers were observed in triple allele mutant cells relative to control cells, the lack of deficiency in *Runx2*, *Col1α1*, and *Tgf-β3* transcripts in triple allele mutant osteoblasts suggests the in vitro bone differentiation defect likely occurs at later stages of differentiation.

Additionally, osteoblast differentiation is modulated by multiple signaling pathways, including the Hh pathway. Mice null for Indian Hedgehog (*Ihh*), a ligand of the pathway, show an absence of osteoblast differentiation [41]. Similarly, *Ift80* conditional knock-out mice show a loss of Hh signaling and impaired osteoblast differentiation [42]. While Hh signaling has been shown to be important in the early stages of osteoblast differentiation by inducing *Runx2* expression [43], the addition of SAG throughout the 21 days of in vitro differentiation enhanced bone nodule formation not only in control cells but also in triple allele mutant cells. This suggests that SAG also enhances later stages of differentiation, countering the triple allele mutant bone formation defect.

Recently, micrognathia in the *ta*^2^ chick mutant was shown to result from incomplete osteoblast differentiation as well as increased bone resorption [44]. An earlier than normal commitment of progenitors to the osteoblast lineage reflected by increased *Runx2* and *Col1α1* in the mandibular prominence of the *ta^2^* chick mutant, similar to what we observed in triple allele mutant osteoblasts in vitro, resulted in incomplete differentiation of osteoblasts, indicated by decreased levels of alkaline phosphatase in the *ta^2^* mandibles of older embryos. Additionally, in *ta*^2^ mandibles, the expression of bone remodeling markers was increased, indicating increased bone resorption. This substantiates that bone resorption levels are inversely proportional to jaw size in avian embryos [45]. Future analyses of bone resorption in triple allele mutant mandibles will be important to determining whether bone resorption and remodeling are critical to establishing jaw length in mammals as well.

Another potential mechanism that may contribute to midface and mandibular hypoplasia are defects in the development of the cranial base. In a comparative study of normal and anencephalic human fetuses, cranial base lengths were reduced in anencephalic fetuses and correlated with maxillary protrusion [46]. Additionally, the cranial base angle negatively correlated with mandibular protrusion. Thus, future analyses of the triple allele mutant cranial base sutures, which form from endochondral ossification, would be informative.

The triple allele mutant craniofacial defects—shortened nasal, mandibular, and midface lengths—appeared quite specific to anterior-posterior measurements. This may be explained if the facial anterior-posterior defects do arise from defects of the cranial base. Another possibility could be that the precise temporal and spatial regulation of signaling pathways by individual ciliary proteins and their interactions regulate the development of specific facial elements [27]. In contrast, other ciliopathy-related craniofacial defects, such as cleft lip and/or palate, hypo-/hypertelorism, broad nasal bridge, craniosynostosis, and facial asymmetry, were not observed, and the development of most facial transverse and vertical dimensions were not disrupted, except for palatal widening. The latter is an interesting finding since palatal width and their growth rates have been proposed to play a role in isolated cleft palate. Siblings of patients with cleft lip and palate have a significantly wider palate than the average population, which may suggest that increased palatal width may increase the risk for cleft lip and palate [47]. Additionally, populations with a higher incidence of cleft lip and palate are associated with broad, short palates, while those with lower rates are associated with narrower, longer palates. Similarly, genetically inbred lines of mice and rats with wider palates and palatal growth rates are at higher risk for cleft lip and palate when exposed to environmental factors in utero [48,49]. Since in mice, the loss of *Thm1* globally or in neural crest cells causes cleft palate [20,27], *Thm1* deficiency may predispose individuals to increased palatal width and the risk of cleft palate.

In summary, this study demonstrates that the genetic interaction between *Thm2* and *Thm1* in mice shapes the postnatal face and presents a novel model to study the etiology of craniofacial ciliopathies.

## Data Availability

Not applicable.

## Supplementary Materials

The following supporting information can be downloaded at: https://www.mdpi.com/article/10.3390/jdb10020017/s1, Table S1: All Measurements with their Corresponding Significance, Table S2: Euclidean and Projected Landmarks.
