# Supplementary material for: Genetic Interaction of Thm2 and Thm1 Shapes Postnatal Craniofacial Bone
Source: J Dev Biol. 2022 May 11;10(2):17. doi: 10.3390/jdb10020017 (PMC9149932; doi:10.3390/jdb10020017)
Supplement: Supplementary file 1 [file jdb-10-00017-s001.zip › jdb-1704260-supplementary.pdf]

Genetic interaction of *Thm2* and *Thm1* shapes postnatal craniofacial bone

Erin E Bumann et al.,

Supplemental Table S1. All Measurements with their Corresponding Significance

Measurement	significant as raw measurement	significant relative to centroid size	Type
**Cranium Centroid Size**	yes	-	**Size**
**Mandibular Centroid Size**	yes	-	
**Snout angle**	yes	-	**Angle**
Mid-anterior cranial vault angle	no	-	
Mid-posterior cranial vault angle	no	-	
Anterior cranial vault angle	no	-	
Facial angle	no	-	
Palate arch angle	no	-	
**Premaxilla length**	yes	yes	**Antero-Posterior**
**Nasal length**	yes	yes	
**Facial region length**	yes	yes	
**Mandibular length (superior)**	yes	yes	
**Mandibular length (inferior)**	yes	yes	
**Upper jaw length**	yes	yes	
**Maxilla length**	yes	no - p<0.057	
**Zygomatic length**	yes	no	
Anterior cranial base length	no	no	
Basisphenod length	no	no	
Presphenoid length	no	no	
Palate length	no	no	
**Palatal width**	no	yes	**Transverse**
**Inter-zygomatic arch width**	yes	no - p<0.053	
**Inter-orbital width**	yes	no	
**Anterior nasal width**	yes	no	
**Inter-maxillary width**	yes	no	
**Inter-(1st)molar width**	yes	no	
Basisphenoid (rostral) width	no	no	
Inter-zygomatic root width	no	no	
Anterior cranial vault width	no	no	
Interior frontal arch width	no	no	
**Mandibular posterior height**	yes	no	**Vertical**
**Ear height**	yes	no	
**Facial height**	yes	no	
Posterior nasal height	no	no	
Anterior pharyngeal height	no	no	
Anterior cranial vault height	no	no	
Frontal crest height	no	no	
Palate height	no	no	

Measurements not shown

Supplemental Table S2. Euclidean and Projected Landmarks

**Landmark Descriptions**	**Measurement type**	**Type**
Midpoint mesial to maxillary incisors	Euclidean	Single
Anterior point at base of nasal aperture	Euclidean	Single
Nasion	Euclidean	Single
Bregma	Euclidean	Single
Lambda	Euclidean	Single
Mid-sagittal point of intersection of interparietal and occipital bones	Euclidean	Single
Medial premaxillary - maxillary junction	Euclidean	Single
Midline point on maxillary-palatal suture	Euclidean	Single
Posterior point on palate	Euclidean	Single
Anterior point on midline of presphenoid (junction with ethmoid)	Euclidean	Single
Intersphenoidal synchondrosis (mid-point of endocranial rostral margin of basisphenoid)	Euclidean	Single
Spine of ethmoidal crest	Euclidean	Single
Mid-point on interior crest of frontal bone	Euclidean	Single
Posterior inferior point on mandibular condyle	Euclidean	Single
Posterior tip of the angular process	Euclidean	Single
Anterior inferior most point on the body of the mandible	Euclidean	Single
Midpoint on alveolar bone lingual to the mandibular incisor	Euclidean	Single
Highest point at the top of the palate	Euclidean	Single
Anterior point on intersection of premaxillary and nasal bones	Euclidean	Paired
Intersection of maxilla, frontal and lacrimal bones	Euclidean	Paired
Anterior-superior point on zygomatic process of maxilla (lateral to infra orbital fissure)	Euclidean	Paired
Anterior end of zygomatic bone	Euclidean	Paired
Posterior end of zygomatic bone	Euclidean	Paired
Anterior point on base of zygomatic process of temporal bone	Euclidean	Paired
Posterior point on base of zygomatic process of temporal bone	Euclidean	Paired
Most prominent lateral point on frontal-parietal suture	Euclidean	Paired
Superior point on interior margin of external auditory meatus	Euclidean	Paired
Inferior point on interior margin of external auditory meatus	Euclidean	Paired
Lateral premaxillary - maxillary suture	Euclidean	Paired
Anterior point on palatine foramen	Euclidean	Paired
Crest of alveolar process (mesial to 1st molar)	Euclidean	Paired
Crest of alveolar process (distal to 3rd molar)	Euclidean	Paired
Posterior point on pterygoid process	Euclidean	Paired
Lateral point of endocranial rostral margin of basisphenoid	Euclidean	Paired
Superior-lateral point on interior crest of frontal bone	Euclidean	Paired
Midpoint mesial to maxillary incisors	Projected	Single
Spine of ethmoidal crest	Projected	Single
Anterior point on midline of presphenoid (junction with ethmoid)	Projected	Single
Spheno-occipital synchondrosis (mid-point of endocranial caudal margin of basisphenoid)	Projected	Single
Intersphenoidal synchondrosis (mid-point of endocranial rostral margin of basisphenoid)	Projected	Single
Posterior point on palate	Projected	Single
Midline point on maxillary-palatal suture	Projected	Single
Nasale	Projected	Single
Nasion	Projected	Single
Highest point at the top of the skull	Projected	Single
Highest point at the top of the palate	Projected	Single
Lateral premaxillary - maxillary suture	Projected	Paired
Anterior end of zygomatic bone	Projected	Paired
Posterior end of zygomatic bone	Projected	Paired

Anterior point on base of zygomatic process of temporal bone	Projected	Paired
Posterior point on base of zygomatic process of temporal bone	Projected	Paired
Superior point on interior margin of external auditory meatus	Projected	Paired
Inferior point on interior margin of external auditory meatus	Projected	Paired
Crest of alveolar process (mesial to 1st molar)	Projected	Paired
Crest of alveolar process (distal to 3rd molar)	Projected	Paired
